# Comparison of the S8 navigation system and the TINAVI orthopaedic robot in the treatment of upper cervical instability

**DOI:** 10.1038/s41598-024-55610-8

**Published:** 2024-03-18

**Authors:** Shuai Li, Jinpeng Du, Yunfei Huang, Dingjun Hao, Zhigang Zhao, Zhen Chang, Xuefang Zhang, Shenglong Gao, Baorong He

**Affiliations:** 1 Department of Spine Surgery, Honghui Hospital, Xi’an Jiaotong University, Youyidong Road, Xi’an, 710000 Shaanxi China; 2 Medical College, Yan’an University, Yan’an, Shaanxi China

**Keywords:** O-arm, 3D real-time navigation system, Tinavi robot, Upper cervical vertebra instability, Diseases of the nervous system, Bone, Trauma, Biomedical engineering

## Abstract

The objective is to compare the clinical efficacy and safety of the S8 navigation system and the Tinavi orthopaedic surgery robot in the treatment of upper cervical instability. The research methods adopted are as follows. The clinical data of patients with upper cervical instability who underwent surgery from May 2021 to December 2021 were analysed retrospectively. Patients were divided into a navigation group (assisted by the S8 navigation system) and a tinavi group (assisted by the Tinavi robot) according to the auxiliary system used. Computed tomography and digital radiography were performed after the operation. The accuracy of pedicle screw placement was evaluated using the criteria put forward by Rampersaud. Degree of facet joint violation, visual analogue scale score, neck disability index and Japanese orthopaedic association score were recorded and assessed during follow-up examinations in both groups. Record two groups of surgery-related indicators. Record the complications of the two groups. A total of 50 patients were included. 21 patients in the navigation group and 29 in the tinavi group. The results of the study are as follows. The average follow-up time was 12.1 months. There was no significant difference in nail placement accuracy between the navigation and tinavi groups (*P* > 0.05); however, the navigation group had a significantly higher rate of facet joint violation than that of tinavi group (*P* < 0.05), and the screws were placed closer to the anterior cortex (*P* < 0.05). Significantly more intraoperative fluoroscopies were performed in the tinavi group than in the navigation group, and the operation time was significantly longer in the tinavi group than in the navigation group (*P* < 0.05). The time of single nail implantation, intraoperative blood loss and incision length in navigation group were significantly longer than those in tinavi group. There were no statistically significant differences in other indicators between the two groups (*P* > 0.05). We come to the following conclusion. The Stealth Station S8 navigation system (Medtronic, USA), which also uses an optical tracking system, and the Tinavi Orthopedic robot have shown the same high accuracy and satisfactory clinical results in the treatment of upper cervical instability. Although the S8 navigation system still has many limitations, it still has good application prospects and is a new tool for spine surgery.

## Introduction

Instability of the upper cervical spine is caused by excessive activity as a result of abnormalities in the bones, joints or ligaments^1^. It is often secondary to inflammation, trauma, congenital, tumour or degenerative diseases and can lead to neck pain and progressive spinal cord injury, which usually requires surgical treatment^2^. At present, the most commonly used methods are posterior cervical pedicle screw fixation and fusion^3^. However, due to large individual differences in the anatomical structure of the upper cervical pedicle and the course of the vertebral artery, the placement of cervical pedicle screws is quite challenging^4^. In traditional surgery, spine surgeons mainly rely on their understanding of the anatomy of the upper cervical vertebra, followed by preoperative and intraoperative fluoroscopic images, to identify anatomical landmarks and verify the correct position of the pedicle screw^5^. However, planar imaging does not provide three-dimensional views or real-time monitoring feedback. In addition, it is necessary to obtain data through the C-arm several times before and after screw implantation, so the operation time is prolonged and the radiation exposure for both patients and operators is increased. Finally, the lower resolution of intraoperative images also makes it difficult for doctors to determine whether the needle entry point is invaded and whether the screw has invaded the surrounding structure. It has been reported that the rate of screw dislocation following bare-handed implantation is 14–23%^6,7^. To improve the accuracy of cervical nail implantation, a variety of intraoperative imaging and navigation systems have been developed. At present, the relatively new systems are the Tinavi Orthopaedic Robot System (codesigned by Beijing Jishuitan Hospital and Tinavi Medical Technologies Co., Ltd.) and the S8 navigation system (Stealth Station S8; Medtronic, Inc., USA), both of which can help surgeons accurately place internal fixation devices^8^.

The Tinavi orthopaedic surgery robot system consists of an image navigation robot positioning platform, including a mechanical arm, an optical tracking system and an operation planning and navigation system, which are directly involved in surgical nail implantation. The robotic arm system has high flexibility and stability and is responsible for surgical planning and path positioning. The optical positioning and tracking system (composed of an infrared stereoscopic camera and reference frame) is responsible for monitoring the relative position of the patient's anatomical structures and the mechanical arm; the operation planning and navigation system collects real-time tracking data, three-dimensional images, and the reference frame and manipulator position reconstructed by O-arm scanning during the operation. Using both the robotic arm and the navigation system allows the effective implementation of the surgical plan. The optical tracking system can detect the actual position and subtle postural changes of the patient in real time and cooperate with the manipulator to compensate for real-time motion so that the manipulator can always be accurately located on the preplanned nail placement trajectory. Finally, the operator uses a guide needle to precisely insert the screw into the pedicle.

S8 navigation system comprises an infrared stereoscopic camera, a navigation reference frame and a navigation workstation. It is a visualization system for computer image processing that provides the necessary data for the operator during nail implantation but does not participate in the specific steps of the operation. The three-dimensional image reconstructed by the O-arm during the operation was used as the carrier, combined with infrared stereo positioning technology to track the anatomical position of the patient and the position of surgical instruments. The anatomical position of the patient and the relative position of the surgical instrument can be detected in real time and dynamically, the nail implantation path can be observed in many directions, the danger zone can be avoided, and the operator can be guided to operate according to the surgical path designed before the operation to achieve high-precision intraoperative navigation. At present, the best auxiliary system for use in the clinical treatment of upper cervical instability is unclear. The purpose of this study was to report the differences in imaging and clinical results between the O-arm imaging combined with the S8 real-time navigation system and the Tinavi orthopaedic robot system in the treatment of upper cervical instability.

## Clinical data and methods

### General information

**Figure 1:**
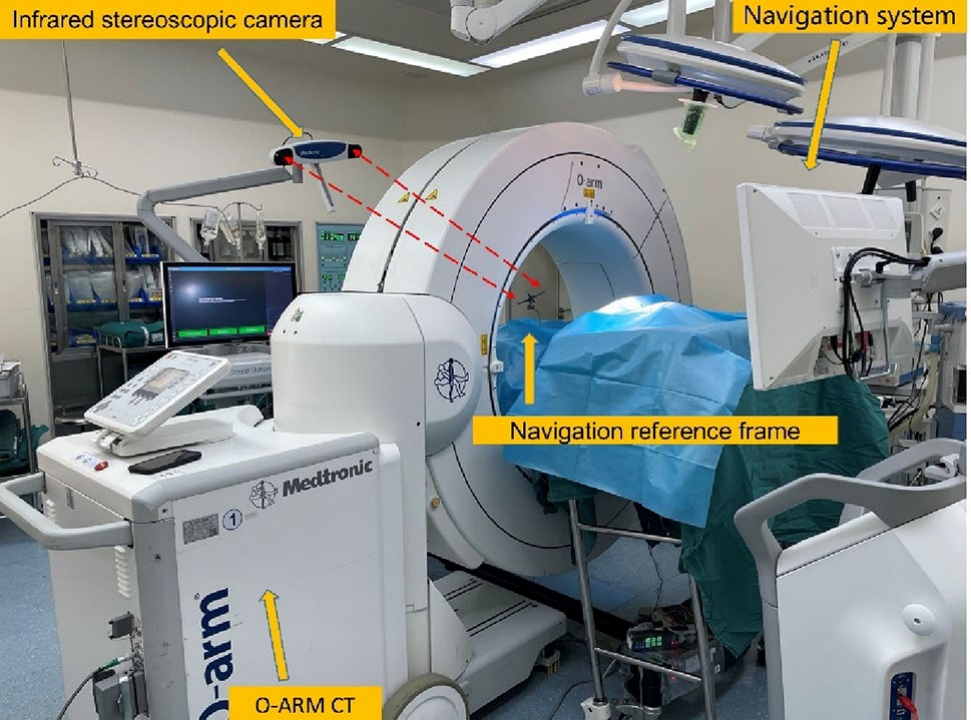
Navigation stereogram.

**Figure 2:**
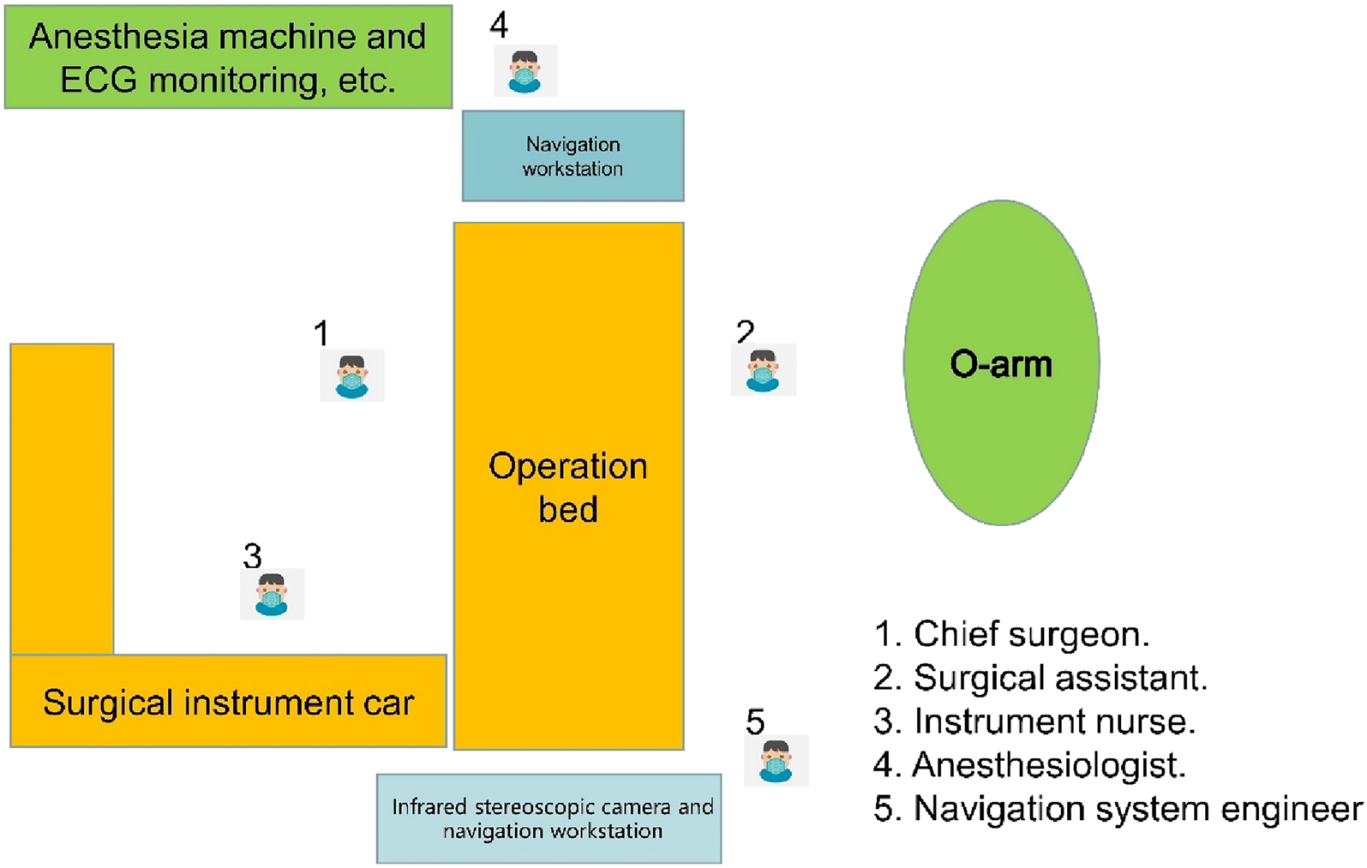
Aerial view of navigation.

The Tinavi orthopaedic surgical robot was introduced in March 2019, and, since then, we have focused on patients who underwent upper cervical pedicle screw fixation assisted by the robotic system. Subsequently, in May 2021, the Stealth Station S8 3D real-time navigation systems was introduced. We collected clinical data related to both device-assisted procedures and performed a retrospective analysis. (Figs. 1 and 2).

All findings were reported in accordance with the Strobe Statement guidelines, and the study was conducted in accordance with the Declaration of Helsinki.

This retrospective study was approved by the Ethics Committee of Honghui Hospital, Xi’an Jiaotong University (approval number: 20230025).

Informed consent was obtained from all subjects and/or their legal guardians.

The inclusion criteria were as follows: (1) patients who need posterior surgery due to atlantoaxial instability caused by non-traumatic factors such as congenital developmental malformation, inflammatory destruction or neoplasms; (2) patients using pedicle screws for internal fixation; (3) patients with complete cervical X-ray, CT and MRI data.

The exclusion criteria were as follows: (1) patients with atlantoaxial instability caused by traumatic fracture, dislocation or subluxation; (2) patients with nerve-related diseases before surgery, or with developmental deformities of the upper cervical vertebrae or an incomplete posterior arch of the atlantoaxial vertebrae; (3) patients with craniocerebral lesions and severe heart, liver or renal dysfunction; severe infection; mental illness; and other conditions; and (4) patients with a history of spinal surgery and cervical disease.

A total of 50 patients were included. All patients were diagnosed with upper cervical instability. All the patients with impaired nerve function in the spinal cord had different degrees of nerve injury symptoms, such as body numbness and decreased muscle strength. Among them, 21 patients were implanted with cervical pedicle screws via the O-arm combined with a CT three-dimensional real-time navigation system (navigation group), and 29 patients were implanted with cervical pedicle screws via the Tinavi robot (tinavi group).

A research assistant was independently responsible for the data collection and follow-up of the patients for at least 12 months.

### Surgical methods

All the operations, including motor-evoked potential (motor-evoked potential, MEP) and somatosensory-evoked potential (somatosensory-evoked potential, SEP), were performed under neuroelectrophysiological monitoring in both groups.

After satisfactory general anaesthesia, the patient was placed prone on the carbon fibre bed, the Mayfield head frame was fixed on the head, and traction was maintained. The upper limbs and shoulders were properly fixed with wide tape, and the surgical area was routinely disinfected and covered with towels after the neuroelectrophysiological monitoring device was installed. A posterior median longitudinal incision was made in the neck, and the length of the incision was determined according to the fixed segments needed for the operation. The layers were separated by layer, and the structures of the posterior arch, vertebral spinous process and lamina, including the atlantoaxial vertebrae, were exposed.

### Navigation group

**Figure 3:**
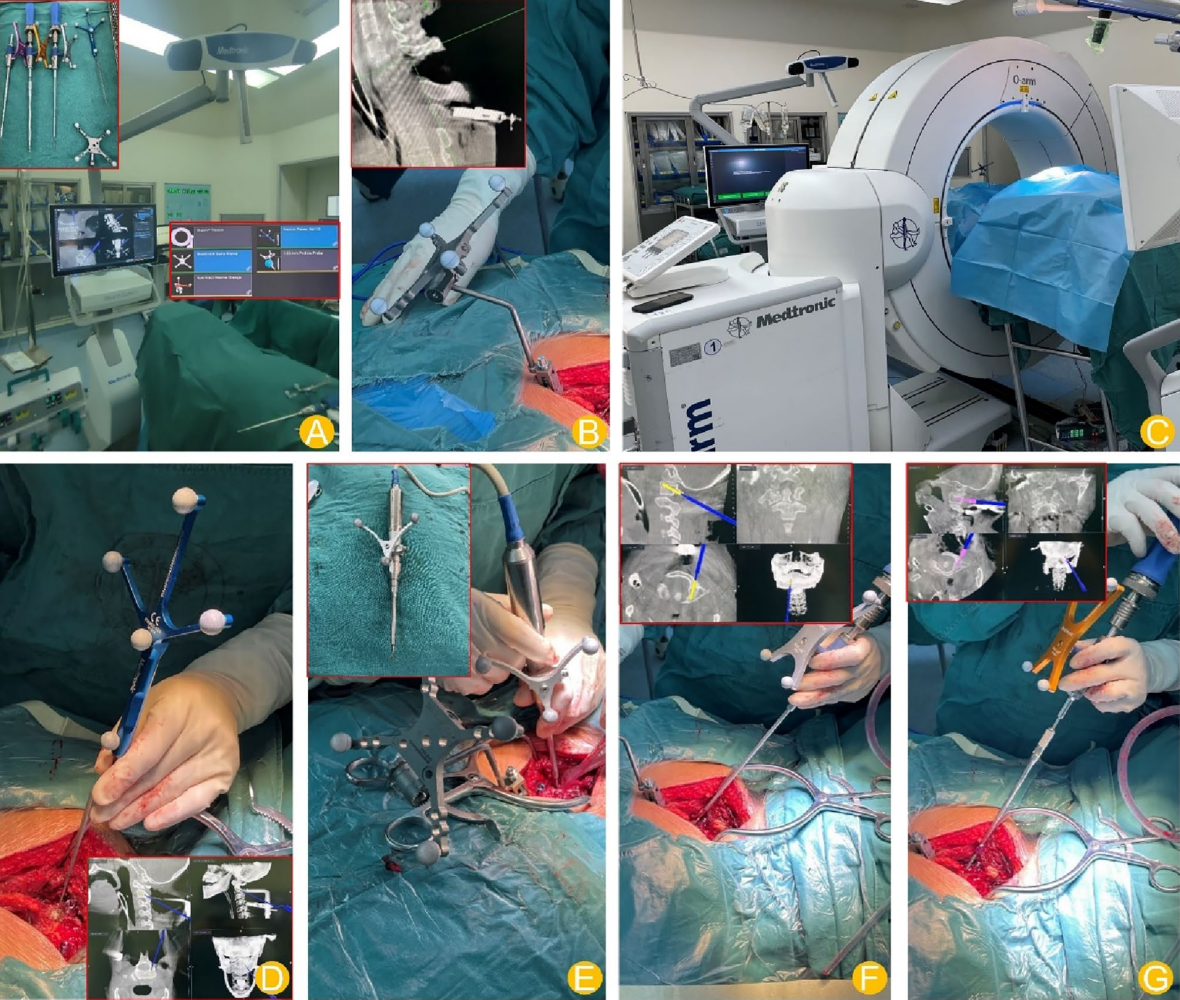
Flowchart.

First, according to the layout of the operating room, the layout of the S8 CT three-dimensional real-time navigation system was planned. Patient information was input, and the navigation reference frame and custom surgical instruments were assembled, matched and verified under the monitoring of infrared stereoscopic cameras (to ensure that the tools are accurate and traceable). In the lower cervical vertebra, a suitable spinous process was selected as the installation point of the navigation reference frame, and the operator placed the navigation reference frame on the spinous process and fixed it properly. After that, the surgical field was covered with a sterile sheet, and the image data were scanned by O-ARM and automatically transmitted to the S8 navigation system. At this time, landmark bone anatomical markers such as the spinous process can be used for calibration. The operator used the navigation probe to determine the appropriate needle entry point, angle and depth of each vertebral body under the guidance of Trajectory 1 and Trajectory 2 interfaces, determined the appropriate screw size and nail placement track, and used a high-speed grinding drill connected with a reference frame to complete the opening. After that, placing screws under the monitoring of the navigation system. During the preparation of the nail path, the navigation probe can be moved in the drill hole many times to observe whether the channel deviates from the proposed track and whether each wall is damaged; additionally, the probe should be adjusted in time if there is any abnormality. Before the next nail path was prepared, the navigation probe was used to contact the patient to verify the bony structure. The navigation system was used to simulate and register each screw, and the operation was completed under full visualization. The implantation of other screws was repeated. Finally, the final image data were confirmed, and the implant location was evaluated. Finally, under conditions of good reduction of the cervical vertebra, a connecting rod of appropriate length and curvature was fixed and locked. After confirming the satisfactory position of the nail rod by fluoroscopy, bone graft fusion was performed, the drainage tube was placed, the incision was closed, and the operation was finished (Fig. 3).

### Tinavi group

First, according to the layout of the operating room, the layout of the Tinavi orthopaedic surgery robot system was planned. The various parts of the Tinavi orthopaedic surgery robot were connected, including the optical tracking system, surgery planning and navigation system and robotic arm system, and the tracer was installed on the bedside bracket. After that, the operation field was covered with a sterile sheet, the patient's spine was scanned in a circle with the surgical vertebral body as the centre by using an O-arm CT machine (Medtronic), and the image data were transmitted to the Tinavi robot worktable to plan the trajectory of the pedicle screws on both sides of the cervical vertebra and control the manipulator to the target area. The guide needle sleeve was inserted to confirm the pedicle entry point, bone cortex at the nail point was ground off with a grinding drill, a pedicle guide needle was inserted along the sleeve with an electric drill, and the manipulator was repeated to insert all the guide needles in turn. A hollow drill was used to enlarge the hole along the direction of the guide needle, after which the pedicle screw was tapped and screwed. Fluoroscopy showed that the screw was in good position. In the same way, the rest of the vertebral bodies were implanted with nails. The subsequent operation was the same as that in the navigation group.

### Postoperative management

Antibiotics and haemostatic drugs were routinely used within 48 h after the operation. Patients were encouraged to wear a cervical brace to get out of bed as soon as possible after the operation, and cervical X-ray images were reexamined. After the patient had worn the neck brace correctly for 3 months, the neck brace was removed, and neck functional exercise began. Regular follow-up was performed 3, 6, 9 and 12 months after the operation. The cervical vertebrae were taken in the positive and lateral positions, and CT scans were performed to evaluate the fusion of the bone grafts.  In addition, The intervertebral height, curvature of the cervical vertebrae and the position of the internal fixation agent were assessed

### Informed consent

Written informed consent was obtained from all subjects involved in the study.

## Main indices

### Accuracy of screw placement

All patients completed the postoperative CT examination, and the postoperative CT image data were measured by the Picture Archiving and Communication System (PACS). The pedicle screw position was independently evaluated by two spine surgeons who were not involved in the operation. Disagreements were settled through negotiation. The accuracy of screw placement was evaluated according to the Rampersaud level^9^. Grade 0: screw completely in the pedicle; grade 1: screw penetration through the pedicle cortex < 2 mm; grade 2: screw penetration through the pedicle cortex < 4 mm; grade 3: screw penetration through the pedicle cortex ≥ 4 mm. A grade of 0 was considered the "ideal screw position", a grade of 0 + 1 was considered "clinically acceptable" screw implantation, and grades 3 were considered "unacceptable" screw placement.

### Invasion of the facet joint violation

The invasion of facet joint violation was evaluated according to the classification system described by Kim^10^. Grade A = no touch, grade B = screw head contact or suspected contact with the facet joint, grade C = obvious screw invasion of the facet joint.

### Secondary indicators

The following clinical data were included: baseline data, operation time, single-nail implantation time, screw density, screw distance from the anterior cortex, intraoperative blood loss volume, intraoperative fluoroscopic dose, incision length, length of hospital stay, VAS score before surgery, three days after surgery, three months after surgery, and at the last follow-up, NDI score before surgery, three days after surgery, three months after surgery, and at the last follow-up, JOA score before surgery, three days after surgery, three months after surgery, and at the last follow-up, and postoperative complications.

### Multivariate analysis

Based on previously published studies, we included confounding factor data that might affect the results of our study, including bmi, bone mineral density, rotation of the vertebral body, wound length and distance between the tracer and operation area. We also conducted a multivariate analysis to determine the influence of these confounding factors on our results and the credibility of our conclusions.

### Statistical analysis

SPSS 25.0 statistical software was used to analyse the data, and the mean ± standard deviation ($\overline{x }$±s) was used to express the measurement data. A t test for two independent samples was used to compare the count data between groups, and the χ2 test was used to compare the count data between groups. The possible factors were included in the multifactor logistic regression, after which the main factors were screened out. The significance level was set at α = 0.05.

## Results

As shown in Table 1, a total of 50 patients met the inclusion criteria; these patients were included in the navigation group (n = 21) and the tinavi group (n = 29). The male-to-female ratio was 31 to 19, the age ranged from 24.95 to 63.39 years, the average age was 44.17 years, and the average bmi was 22.04. A total of 84 screws were implanted in the navigation group, and 116 screws were implanted in the tinavi group. The main diagnosis was upper cervical instability. There was no significant difference in the baseline data between the two groups (*P* > 0.05).

**Table 1:** Comparison of preoperative general information between the two groups of patients.

Index		Navigation group	Tinavi group	t/Z/χ^2^	*P*
		N = 21	n = 29		
Sex	Male Female	13 8	18 11	0.00	0.990
Age (years)		44.17 ± 19.22	45.23 ± 16.88	0.21	0.837
BMI		21.46 ± 2.7	22.61 ± 1.99	1.74	0.089
Diseases	Free odontoid process	5	6		
	Occipitocervical complex deformity	4	8		
	Rheumatoid arthritis	6	6	1.17	0.882
	Degeneration	3	3		
	Tumour	3	6		
Neurological status (ASIA grade)	Abnormal	17	24	0.03	0.869
	Normal (Grade E)	4	5		

### Accuracy of pedicle screw placement and facet process invasion

The accuracy of the implantation of "perfect" and "clinically acceptable" pedicle screws in the navigation group was 89.29% and 95.24%, respectively, whereas that in the tinavi group was 90.52% and 95.69%, respectively. Moreover, there was no significant difference between the two groups (*P* > 0.05). With regard to facet joint violation, there were significant differences between the navigation group (A–C: 96.48%, 9.52% and 0%) and the tinavi group (A–C, 97.41%, 2.59% and 0%) (*P* < 0.05) (Table 2).

**Table 2:** rating of implanted screws and comparison of facet joint violation.

Index	Navigation group	Tinavi group	t/Z/χ^2^	*P*
Screw rating				
0 level	75	105	0.08	0.775
1 level	5	6		
0 + 1 level	80	111	0.02	0.879
2 level	4	5		
3 level	0	0		
Invasion of facet joint violation				
A	76	113	4.51	0.034
B	8	3		
C	0	0		

Grade 0: the pedicle screw is completely in the pedicle.

Grade 1: screw perforation of the pedicle cortex < 2 mm.

Grade 2: screw piercing pedicle cortex ≥ 2 mm and < 4 mm.

Grade 3: the screw penetrated the pedicle cortex ≥ 4 mm (or the screw deviated completely).

A grade of 0 was considered the ideal screw position, a grade of 0 was considered to indicate clinically acceptable precision, and a grade of 3 was considered unacceptable.

Grade A, without proximal joint invasion; Grade B, with proximal articular surface invasion < 1 mm; and Grade C, with proximal articular surface invasion.

### Clinical results

**Figure 4:**
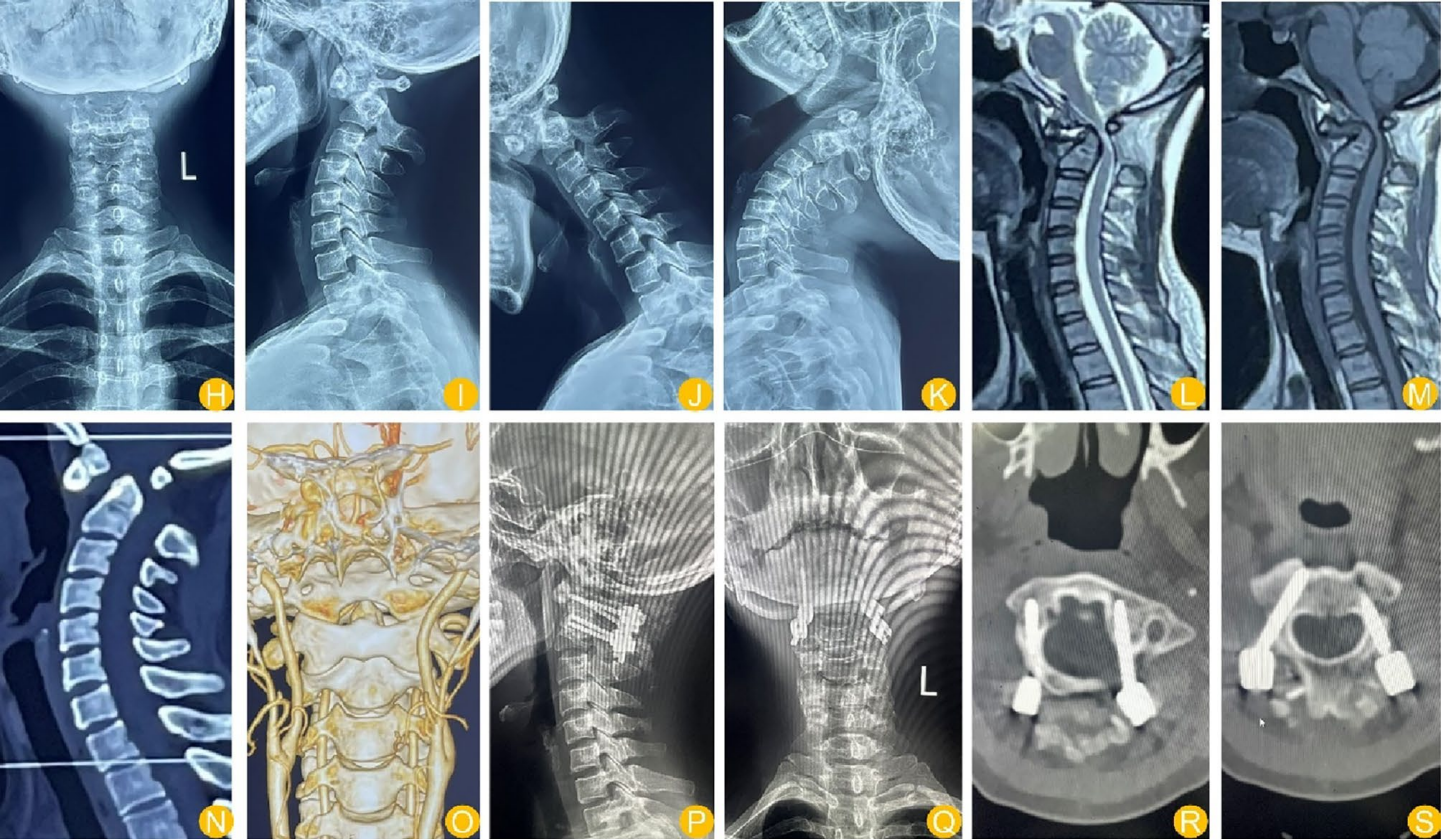
Navigation group. The patient, a female, was admitted to the hospital for a 1-year history of numbness and weakness in the extremities and a 3-month history of unsteady walking. The patient had a muscle strength grade of 4 and positive pathological signs, underwent elective surgery, and recovered well after surgery. (H–K): Preoperative anterolateral and hyperextension and hyperflexion DR (L–M): Preoperative MRI (N–O): Preoperative CT (P–Q): Postoperative DR (R–S): Postoperative CT.

**Figure 5:**
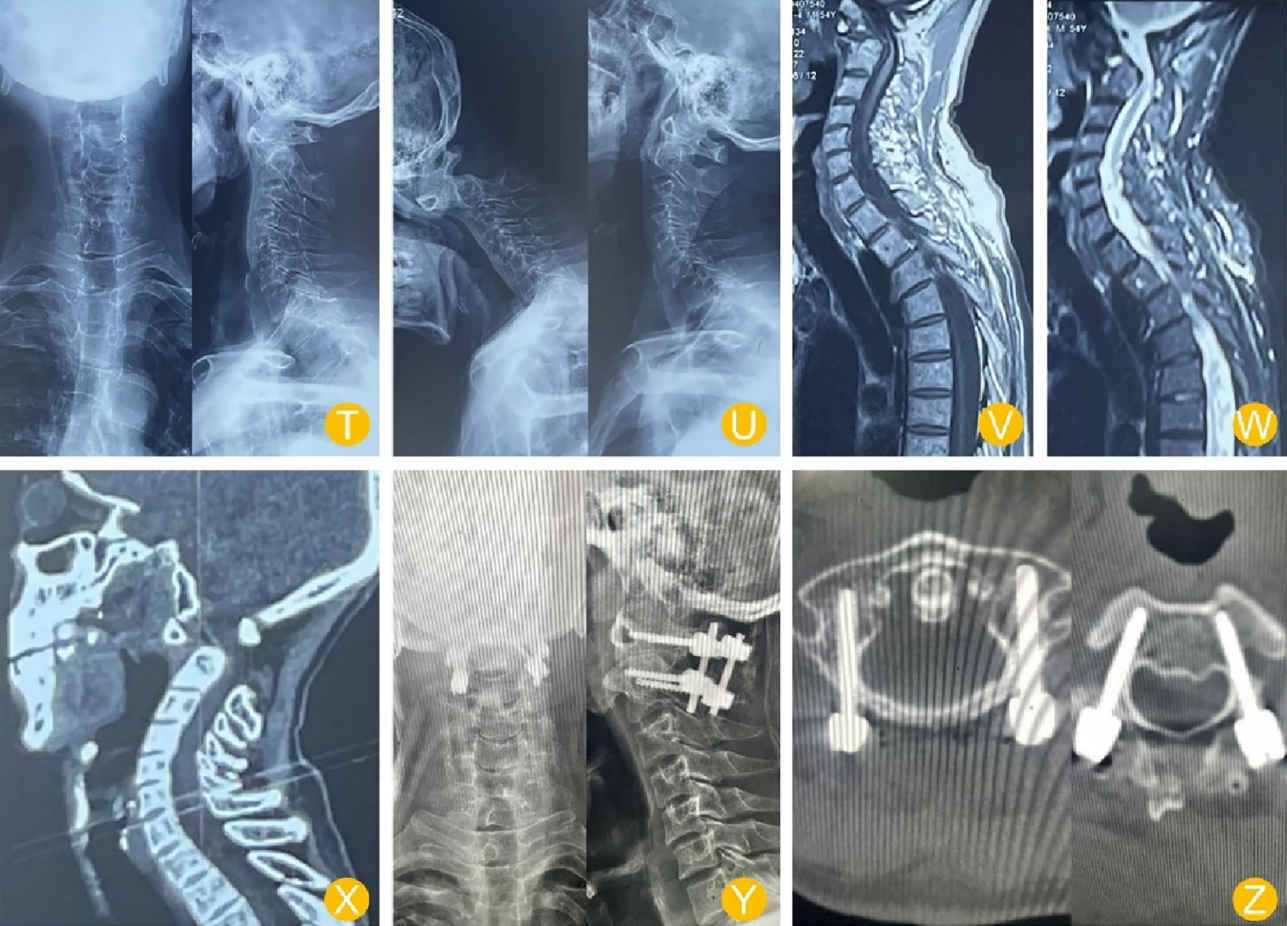
Tinavi group. The patient was male and admitted to the hospital for a 3-month history of numbness and weakness in the extremities, unstable walking; he had a muscle strength grade 3 and positive pathological signs; he underwent elective surgery and had good postoperative recovery. (T–U): Preoperative anterolateral and hyperextension and hyperflexion. DR (V–W): Preoperative MRI. (X): Preoperative CT. (Y): Postoperative DR. (Z): Postoperative CT.

All patients were followed for an average of 12.1 months. The time of single-nail implantation (from the skin to the completion of implantation), intraoperative blood loss volume and incision length were greater in the navigation group than in the tinavi group (*P* < 0.05). However, the number of intraoperative scans, fluoroscopic dose and operation time were greater in the tinavi group than in the navigation group (*P* < 0.05). Moreover, there were no significant differences in other clinical parameters, such as screw density, length of hospital stay, JOA score, NDI score or VAS score, between the two groups. The distance from the screw to the cortex was shorter in the navigation group than in the tinavi group (*P* < 0.05). There were no serious complications, such as screw loosening or infection, between the two groups, and there was no significant difference between the two groups (*P* < 0.05) (Tables 3, 4, 5 and 6) (Figs. 4 and 5).

**Table 3:** comparison of intraoperative and operative related indices between the two groups.

Index	Navigation group	Tinavi group	t/Z/χ^2^	*P*
Operation time (min)	201.00 ± 42.67	232.63 ± 60.33	2.056	0.045
Single-nail implantation time (min)	3.69 ± 0.66	2.51 ± 1.11	4.693	0.000
Screw density	88.10 ± 7.99	87.71 ± 8.14	0.168	0.867
Distance from screw to anterior cortex (mm)	5.77 ± 1.82	9.69 ± 1.05	8.860	0.000
Bleeding volume (ml)	260.19 ± 60.49	200.84 ± 43.66	4.034	0.000
Fluoroscopic dose (mGy)	366.12 ± 79.86	463.15 ± 75.46	4.379	0.000
Notch length (cm)	7.26 ± 0.96	4.99 ± 1.11	7.540	0.000
Length of stay (days)	5.23 ± 2.31	5.33 ± 2.17	5.619	0.876

Definition of single-nail implantation time: After full exposure, the needle entry point was determined from the beginning to the end of nail placement.

**Table 4:** VAS score.

	Navigation group	Tinavi group	t/Z/χ^2^	*P*
Before operation	6.11 ± 0.49	6.2 ± 0.6	0.564	0.575
3 days after operation	3.19 ± 1.19	3.12 ± 1.28	0.196	0.845
3 months after operation	1.70 ± 0.88	1.6 ± 0.7	0.447	0.657
Last follow-up	1.55 ± 0.20	1.5 ± 0.25	0.757	0.453
*P*	< 0.001^a^	< 0.001^a^		

^a^indicates that there was a significant difference before and after the operation.

**Table 5:** JOA score.

	Navigation group	Tinavi group	t/Z/χ^2^	*p*
Before operation	7.57 ± 1.99	7.62 ± 2.11	0.085	0.933
3 days after operation	11.72 ± 2.18	11.66 ± 2.72	0.083	0.934
3 months after operation	14.55 ± 2.19	14.64 ± 2.66	0.127	0.900
Last follow-up	16.33 ± 2.61	16.21 ± 2.5	0.164	0.870
*P*	< 0.001^a^	< 0.001^a^		

^a^represents a significant difference between postoperation and preoperation.

**Table 6:** NDI score.

Index	Navigation group	Tinavi group	t/Z/χ^2^	*p*
Before operation	30.26 ± 2.99	31.5 ± 2.7	1.532	0.132
3 days after operation	19.66 ± 1.96	20.14 ± 1.92	0.865	0.391
3 months after operation	13.11 ± 1.62	12.86 ± 1.54	0.484	0.630
Last follow-up	10.99 ± 1.98	11.4 ± 2.4	0.640	0.525
*P*	< 0.001^a^	< 0.001^a^		

^a^represents a significant difference between postoperation and preoperation.

### Multivariate analysis

According to the findings of this study and previous literature, we included data on confounding factors that may affect the results of the main research indicators, including BMI, bone mineral density, rotation of the vertebral body, wound length and distance between the tracer and operation area. Multivariate analysis was also conducted to determine the impact of these confounding factors on the credibility of the results and conclusions. As shown in Table 7, BMI (*p* = 0.023; OR 2.301 95% CI 1.087–4.634) and bone mineral density (*p* = 0.031; OR 2.417 95% CI 1.881–3.421) were potential confounding factors.

**Table 7:** Multifactorial analysis.

Variable	Regression coefficient	Standard error	Wald value	*P*	OR	95% CI
BMI	3.271	1.101	3.092	0.023	2.301	1.087–4.634
Bone mineral density	2.232	1.108	4.317	0.031	2.417	1.881–3.421
Rotation of vertebral body	2.011	0.846	4.287	0.085	2.314	0.902–4.534
Wound length	3.804	1.831	4.411	0.782	3.211	0.862–5.311
Distance between tracer and operation area	3.211	1.101	5.306	0.634	4.393	0.524–9.520

## Discussion

The main goals of surgery for upper cervical instability are to restore a normal anatomical relationship, reconstruct stability, minimize surgical trauma, reduce postoperative complication rates and improve long-term effects. However, because the pedicle of the upper cervical vertebra is adjacent to important anatomical structures, such as the vertebral artery, nerve root and cervical spinal cord, it is more difficult to place nails if the upper cervical spine is complicated by congenital deformities and degenerative changes. If the screw penetrates the pedicle cortex, it may cause some irreparable neurological complications^11^. As the clinical requirements for improving the accuracy of nail implantation require continuous improvements in nail implantation technology, a variety of auxiliary nail implantation systems have been used in the clinic to solve the above problems. In our study, we found that, compared with the Tinavi orthopaedic surgical robot, the S8 navigation system allows real-time dynamic visualization; is simple and convenient to operate; has a short operation time; and stronger screw holding force (Table 8).

**Table 8:** The technical aspects of the S8 navigation system and Tinavi orthopaedic surgery robot.

	S8 navigation system	Tinavi orthopaedic surgery robot
Anatomic race based	Foreign country	home
Whether he was directly involved in the surgery	NO	YES
Image-based data	O-arm	O-arm
Whether it has an optical positioning and tracking system	YES	YES
Whether the device can be customized	YES	NO
Whether there is a robotic arm	NO	YES
Whether to verify after nail placement	YES	YES

By analysing the working steps and principles of the two systems, we found that although the specific operating steps are quite different and play different roles during surgery, compared with other nail implantation auxiliary systems, both have the greatest ability to monitor the anatomical structure of patients and the relative spatial position of surgical instruments. This function comes from the common component of both-infrared stereoscopic cameras. This can also explain the high accuracy of both methods. As a result, we can imagine that adding an infrared stereoscopic camera to any orthopaedic auxiliary system can also improve the accuracy of nail implantation. Because the nail path implantation process of the S8 navigation system is similar to the traditional manual nail path implantation process, it is prone to invasion and facet joints; moreover, the duration of single-nail implantation is longer than that of the Tinavi orthopaedic robotic implantation. However, due to the frequent need for repeated registration of the Tinavi robot and the lack of intraoperative real-time detection, the total operation time was longer than that of the navigation group because the screw accuracy is frequently verified by an X-ray machine. Because S8 navigation can help surgeons detect more advanced real-time positions of screws, they can be closer to the anterior cortex during screw implantation, and a more reliable holding force can be obtained. The accuracy of the implantation of "perfect" and "clinically acceptable" pedicle screws in the navigation group was similar to that of others^12^, and that of the Tinavi group was similar to that reported by others^13^. Moreover, there was no significant difference between the two groups. Because both collect data through the O-arm, the amount of radiation used is higher than that used for traditional manual nail implantation, which is one of the disadvantages that cannot be avoided by any other nail implantation methods at present. The tinavi group usually required more frequent registrations, so the radiation dose was higher; however, the navigation group had a shorter scanning time, did not need multiple scans and had a lower radiation dose. For operators, exposure to radiation is almost zero because of their timely leave before image acquisition. Finally, because the reference frame of the tinavi group could be placed on the bedside column, the required incision was shorter than that of the navigation group. Moreover, there were no significant differences in other clinical follow-up results, such as the NDI, JOA, or VAS score.

With the in-depth understanding of navigation systems, there are many suitable applications for S8 navigation systems. Posterior cervical approach surgery often causes axial pain, which is mainly caused by extensive muscle peeling and the wound healing process^14^. Therefore, the development of minimally invasive technology is highly important for reducing bleeding, perioperative morbidity, surgical pain and promoting postoperative recovery. However, these procedures are associated with a higher risk of neurovascular dural injury. With precise planning via S8 navigation, trauma and the complication rate can be effectively reduced, especially for patients in poor physical condition^15^. Second, in kyphoplasty, S8 navigation can avoid possible pedicle stenosis, variation and severe vertebral compression fractures, and the operator can be instructed to plan a predetermined track to reach the ideal puncture point, which makes the distribution of bone cement more uniform and prevents a series of complications caused by uneven distributions of stiffness and hardness in the vertebral body. This approach not only yields the most ideal surgical effect but also reduces the risk of bone cement leakage and structural damage near the puncture point. Similarly, S8 navigation can also be used in disease diagnosis; it can accurately puncture the focus and facilitate multipoint sampling to improve the positive rate, reduce the damage to the surrounding structure, and accurately plan the focus in the follow-up focused debridement link. It can not only achieve the best debridement effect but also control the destruction of the normal structure. Finally, in traditional preoperative planning, doctors can customize an operation plan by only analysing a large number of 2D images, with the help of clinical experience; however, there are a large number of complex and important structures around the spine. It is difficult to mentally reconstruct the three-dimensional spine before surgery, which can easily cause the preoperative plan to be imperfect and thereby increase unnecessary radiation exposure. This requires that clinicians have a large amount of systematic and standardized training and practice. More objective, accurate and intuitive three-dimensional visual images generated by the S8 navigation system can be used to reconstruct the immediate effect of intraoperative nail placement from multiple angles, which is helpful for understanding anatomy. With the help of navigation, surgeons can precisely place the nail, which can reduce the tension associated with difficult nail placement and relieve fatigue during the operation. It is highly important to guide novices and enrich their experience.

Despite its many advantages, the S8 navigation system still has many disadvantages. (1) At present, the use and maintenance of the S8 navigation system are expensive, but studies have shown that in hospitals in which more than 250 navigation spinal surgeries are performed each year, this technique is cost effective because it reduces complications and revision rates^16^. Additionally, the S8 navigation was developed by foreign staff members, and foreign surgical instruments are used as adaptation objects and have poor adaptability. Once a system fails, nails need to be implanted by hand, which increases the risk of the operation, and helping grassroot hospitals is difficult. (2) During the navigation operation, the placement of the reference frame is unstable or mistakenly touched, the visual field of the infrared stereoscopic camera is blocked, the navigation marker ball is easily contaminated, and it is difficult to always face the infrared stereoscopic camera during operation, which often leads to a lag in the real-time position display of surgical instruments and increases the risk of screw misplacement. (3) Due to the steep learning curve, the operation time may be prolonged before the surgeon becomes proficient. (4) When obtaining O-arm CT data, the anaesthesiologist should cooperate with the anaesthesiologist to reduce the tidal volume or suspend mechanical ventilation to avoid errors caused by thoracic movement during mechanical ventilation, which increases the risk of the operation. (5) A decrease in the accuracy and invasion of the facet process caused by human function cannot be avoided. (6) At present, the size of the S8 navigation device is large, but the volume of the operating room is generally small. Surgeons in close contact with patients need strict aseptic treatment, and patients are more likely to be infected during such procedures. (7) The use of more advanced imaging equipment to achieve higher image resolution and more accurate location is usually accompanied by increased radiation exposure. (8) Multistep preparation of the nail path is needed when the nail is implanted. Because different operators have different experiences in nail placement, each operator's three-dimensional judgement and proficiency during surgery strongly influence the accuracy of nail placement in the process of adjusting and determining the nail path. (9) Although the patients were fixed with a head frame before the operation, the possibility of cervical motion cannot be ignored. Even if part of the vertebral body moves after the release of cervical paraspinal muscles or if the force is not applied properly during the process of nail implantation, the relative position of the vertebral body may be changed, resulting in a decrease in the accuracy of nail implantation. (10) Due to metal artefacts found in revision patients, intraoperative CT data may contain abnormal information and poor quality images^9^.

In view of the above many application defects, we suggest the following: first, for experienced cervical pedicle screw implantation, the operation time can be greatly reduced by manual nail implantation combined with the S8 navigation system. Second, the position of the navigation reference frame should be reasonable; otherwise, it will lead to inaccurate placement. Several studies have shown that placing the reference frame in the skin increases the accuracy of placement, and once the reference frame is loose, it needs to be reregistered; after registration, the navigation probe is used to contact the obvious bone marks in the operation area to verify the accuracy of the navigation guide needle. If it is suspected that the operation may lead to changes in anatomical structure during the operation, the pedicle screw should be checked with fluoroscopy in a timely manner, and re-register should be used if necessary to improve the accuracy of navigation^17^. Third, the infrared stereoscopic camera should be directly connected to the reference frame and the line of sight of surgical instruments. The surgical team was streamlined, and all personnel were familiarized with the sterile area. The monitor should be placed in a position that allows the operator and assistant to have clear visibility to reduce the error due to distraction caused by the navigation display. It has been reported that with the increase in the number of registered vertebral segments and cervical pedicle insertion groups, the dislocation rate of cervical pedicle screws increases significantly, while the dislocation rate of cervical pedicle screws decreases when performing separate registration. Therefore, when the cervical spine has a large range of motion, it is best to use separate registration to reduce the error. Care should be taken in the process of nail implantation to avoid errors caused by vertebral movement caused by improper application of force. Finally, to minimize the radiation dose used in patients receiving O-arm therapy, Su proposed that the use of a paediatric fluoroscopic dose of O-arm can reduce the radiation dose to the normal level of 1 to 4, and the image quality can still guide screw implantation and has no significant effect on the accuracy of screw placement^18^. Several scholars have also proposed that after the end of screw placement, pedicle screw-evoked EMG signals are used to determine the screw position; if the EMG signal is not abnormal, there is no need for O-arm three-dimensional scanning after screw placement^19^.

According to our multivariate analysis, only bone mineral density and BMI were identified as potential confounding factors. A BMI ≥ 28 kg/m^2^ is a risk factor for navigation system-assisted nail placement, probably because the soft tissue of obese patients is too thick and tissue tension is too high. However, the average BMI of the patients in this study was low. The bone quality of some patients with osteoporosis (- 3.5 < T ≤ − 2.5 or T ≤ − 3.5) was poor. Although navigation system-assisted screw placement can accurately guide the screw entry point, some screws cannot be correctly fixed to the bone, which may lead to screw deviation. In this study, the patients were younger and had better bone mineral density. Overall, confounding factors had no significant impact on the conclusions of this study.

This study has several limitations. First, there is bias in this single-centre retrospective comparative study, which affects the conclusions. Second, because we are still in the early stage of use, the number of patients included was insufficient, so we need to continue to increase the number of patients in the later stage to increase the credibility of the research conclusions. Third, retrospective research methods will inevitably lead to the loss of clinical data; however, additional clinical data need to be obtained in further forward-looking studies combined with more comprehensive evaluation indicators to analyse the conclusions. Therefore, longer follow-up, randomized case‒control and multicentre studies are needed in the future.

## Conclusion

Compared to the Tinavi orthopaedic robot system, the O-arm combined with the Stealth Station S8 navigation system (Medtronic, Inc., USA), which also uses an optical tracking system, has the same high accuracy and satisfactory clinical efficacy in the treatment of upper cervical instability. Although the S8 navigation system still has many limitations, it still has good application prospects and is a new tool for spinal surgery.

## Data Availability

The datasets generated and/or analysed during the current study are available from the corresponding author upon reasonable request.
